# Recurrent stroke in an African female with idiopathic thrombotic thrombocytopenic purpura: A case report

**DOI:** 10.1002/ccr3.6860

**Published:** 2023-01-16

**Authors:** Kwabena Oteng Agyapong, Aba Folson, Roland Wonkyi, Kelvin Amenyedor, Jeffrey J. Boateng, Kate Fiador

**Affiliations:** ^1^ Department of Internal Medicine Greater Accra Regional Hospital Ridge – Accra Ghana; ^2^ School of Medicine University of Health and Allied Sciences Ho Ghana; ^3^ Eastern Regional Hospital Koforidua Ghana; ^4^ Department of Haematology Greater Accra Regional Hospital Ridge – Accra Ghana

**Keywords:** ADAMTS 13, MAHA, platelet, thrombotic thrombocytopenic purpura

## Abstract

We report on a young Ghanaian female who was diagnosed with thrombotic thrombocytopenic purpura (TTP) but had an ischemic stroke as the initial presentation. She was successfully treated with therapeutic plasma exchange. This case illustrates how TTP can masquerade as ischemic stroke and the application of PLASMIC score without ADAMTS‐13 assay in risk prediction.

## 1 INTRODUCTION

Thrombotic thrombocytopenic purpura (TTP, Moschcowitz disease) is a triad of severe thrombocytopenia (usually less than 100 × 10^9^/L), macroangiopathic hemolytic anemia, and varying end‐organ involvement.^1^ TTP occurs following the increase in prothrombotic state with the deficiency in the disintegrin and metalloproteinase with thrombospondin motifs 13 (ADAMTS 13), the molecule that cleaves and thereby activates the von Willebrand factor (vWF).^2^ Large, uncleaved multimers of vWF form a substrate for the binding of platelets within the blood vessels with subsequent mechanical fragmentation of red blood cells, occlusion of the microcirculatory bed, and organ ischemia. The more prevalent form of this disease is acquired, with the formation of autoantibodies against ADAMTS 13, however, compound heterogeneous or homogeneous mutations may also occur: Upshaw‐Schulman syndrome.^3^, ^4^ TTP usually presents acutely with rapid deterioration of the patient and has a high mortality and recurrence rate in those who survive an acute episode. Neurologic (headache, seizures, and coma), cardiac (ST elevation and non‐ST elevation myocardial infarction, heart failure), and renal manifestations have been documented and many patients develop multiorgan failure leading to their eventual demise within weeks if not adequately managed.^5^, ^6^ The bedrock of treatment is the use of therapeutic plasma exchange with fresh frozen plasma as this reduces the autoantibodies against ADAMTS 13 and improves the levels of functional vWF.^7^

## 2 CASE REPORT

**FIGURE 1:**
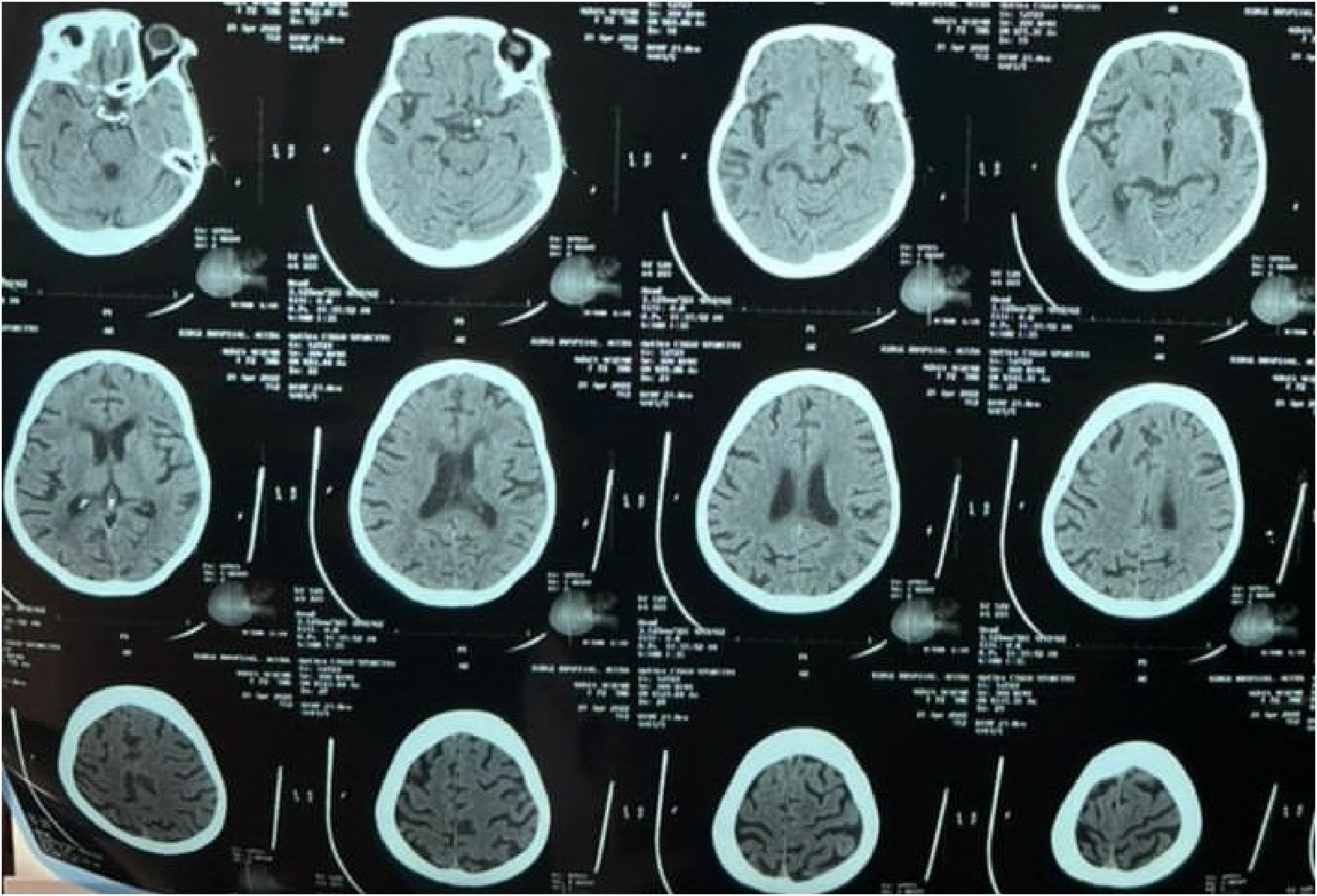
CT scan of the head: initial head CT scan on presentation and admission

A 40‐year‐old African female was referred to the Emergency on account of a stroke confirmed by a CT scan brought from the referring center (Figure 1). She presented with slurred speech, facial asymmetry, and left upper and lower limb weakness of 1‐day duration. The patient had been diagnosed with hypertension 10 years ago but had been noncompliant with her medication 2 weeks before her presentation. Her medications included amlodipine and lisinopril. She admitted to headaches, dizziness, palpitations, and easy fatigue and reported menorrhagia for the past 10 years. Past medical history included two stroke events 5 years ago and encephalitis at age 8 months. There was a family history of hypertension and diabetes but no history of bleeding disorder.

On physical examination, she was neither in respiratory distress nor jaundiced, however, was severely pale. She had a purpuric patch on the flexural areas of both elbows and knees and the posterior aspect of the lower legs. The patient was afebrile with a temperature of 36.8°C, pulse rate of 81 beats per minute, respiratory rate of 20 cycles per minute, and blood pressure of 103/67 mmHg at presentation. Power grading in her left upper and lower limbs was 1/5. All other systems were unremarkable.

The complete blood count revealed a low hemoglobin level of 4.3 g/dl (11.5–14.5), platelet count of 18 × 10^9^/L (150–450 × 10^9^/L), white cell count of 10.47 × 10^9^/L, and a normal renal function. The initial working diagnosis was an acute ischemic stroke with left hemiparesis, Abnormal uterine bleeding from a uterine leiomyoma, hematological malignancy, and a bleeding diathesis. Samples were taken for blood film comment, blood film for malaria parasite, LDH, uric acid, clotting profile, HIV, ANA, and Hepatitis B and C. Additionally, an electrocardiogram, echocardiogram, abdominopelvic ultrasound, and chest X‐ray were requested. She was transfused with 1 unit of whole blood and neurological physiotherapy started. On days 2 and 3 of admission, her power improved to 4/5.

**FIGURE 2:**
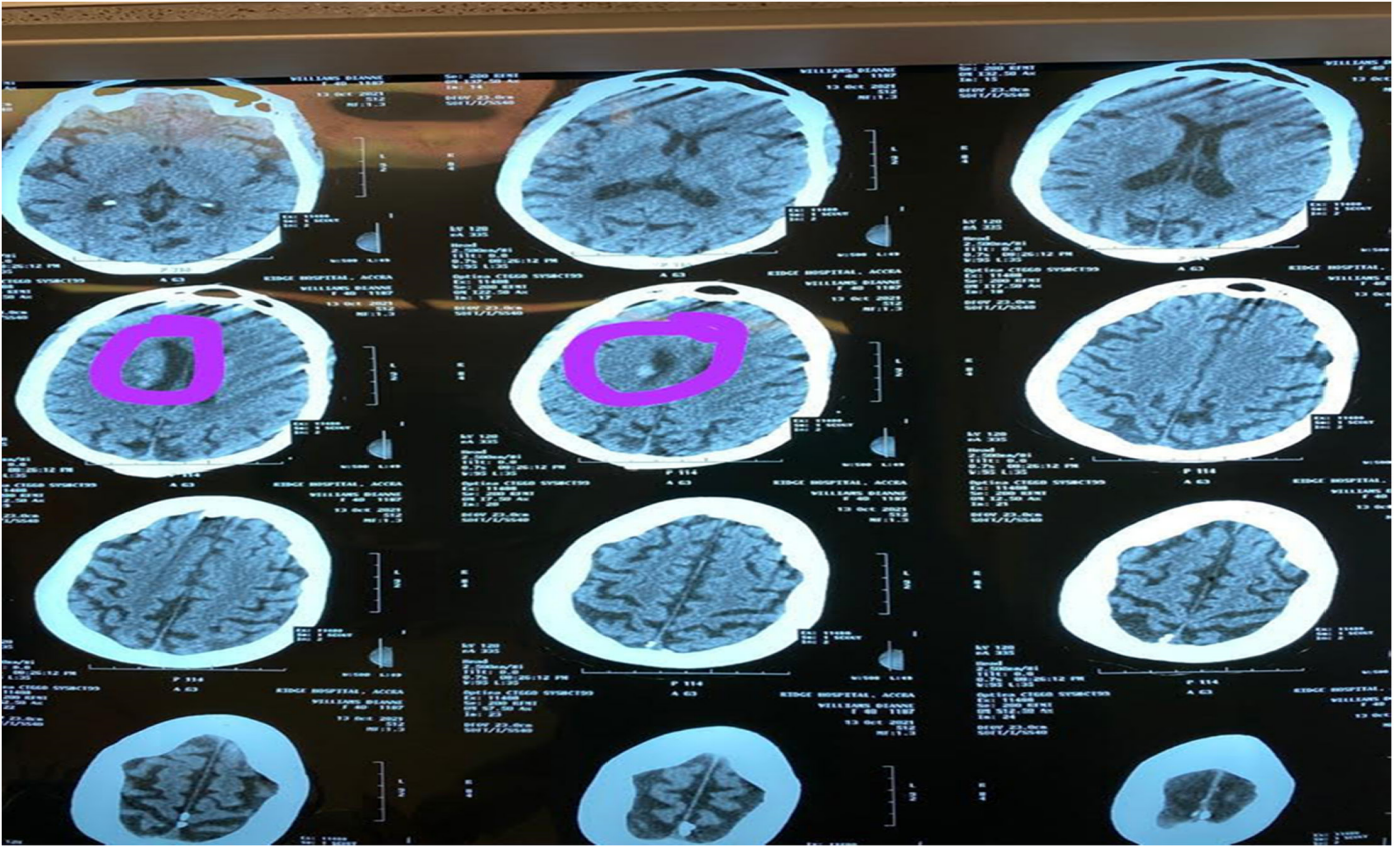
CT scan of the head taken during recurrent episodes of seizures

On day 4, the patient developed multiple episodes of tonic–clonic seizures and she gradually developed jaundice the same day. Repeat complete blood count showed Hb of 6.2 g/dl, WBC of 13.5 × 10^9^/L, and platelet count of 22 × 10^9^/L. Clotting profile results showed an INR of 1.16, PT – 9.8 s, APTT – 33.9 s, and PTT – 0.92 s. LDH was 2.6111 μ/L (135–214), Uric acid was 0.4 mmol/L (0.13–0.39), and both direct (19.86 μmol/L) and indirect (34.7 μmol/L) bilirubin were high with low globulin levels (25.7). HIV, Hepatitis B and C, ECG, chest X‐ray, blood film for malaria parasite, AST, ALT, AST, and ANA were all normal. A thyroid function test was normal. The working diagnosis was then modified to recurrent seizures with a differential of a recurrent stroke with hemorrhagic transformation, poststroke seizures, intracranial infections such as meningoencephalitis, electrolyte encephalopathy, and intracranial hemorrhage from idiopathic or acquired thrombocytopenia. A repeat head CT scan (Figure 2) showed a periventricular bleed. The result of the blood film comment showed normocytic normochromic RBCs, schistocytes ++ with occasional normoblast, few polychromatic cells, increased WBC count mostly neutrophils with a left shift, and reduced platelets with no clumps seen suggestive of microangiopathic hemolytic anemia. ADAMTS‐13 assay could not be conducted as requested due to inadequate funds. Alternatively, the TTP PLASMIC score was used for confirmation which yielded a total score of 7 (high risk) and a 96.2% risk of severe ADAMTS13 deficiency (≤10%). A repeat of the renal function test yielded urea of 31.7 mmol/L (2.1–7.1) and creatinine of 492 μmol/L (62–106). TTP was diagnosed, and the patient was transfused with 3 units of whole blood, 4 units of fresh frozen plasma, and 3 days of pulsed methylprednisolone. Subsequently, she had a total of 7 sessions of plasmapheresis and 3 sessions of hemodialysis with concomitant oral prednisolone 80 mg and 1 g of 10% calcium gluconate before plasmapheresis. Her GCS at the end of the sessions rose to 15/15. The patient was not pregnant at any time during her illness (Tables 1 and 2). The first plasmapheresis was done on 17/10 and hemodialysis on 18/10.

**TABLE 1:** Summary of patient's CBC laboratory reports before, during, and after plasmapheresis and hemodialysis

CBC parameter	Date (dd/mm) from admission to the last review															
	7/10	9/10	11/10	14/10	16/10	17/10^a^	18/10	19/10	21/10	23/10	24/10	26/10	27/10	30/10	4/11	9/11
Hb (g/dl)	4.3	6.2	8.7	6.6	4.7	8	8.5	6.7	9.7	9.9	7.7	8.8	8.5	6.5	6.3	9.7
WBC (×10^9^/L)	10.5	13.5	8.2	22.5	9.5	9.3	12.7	8.2	9.7	11.6	10	23	22.3	22.7	6.31	6.21
Platelet (×10^9^/L)	18	22	47	45	39	20	28	47	59	83	94	52	33	43	149	174

^a^ Date of the first plasmapheresis.

**TABLE 2:** Summary of patient's BUE and CR laboratory reports before, during, and after plasmapheresis and hemodialysis

BUE, CR parameter	Date (dd/mm) from admission to the last review					
	7/10	18/10^a^	21/10	25/01	4/11	9/11
Na (mmol/L)	135	144	138	138	147.7	141
K+ (mmol/L)	3.97	3.7	4.9	4.1	3	3.5
Cl‐ (mmol/L)	100.1	109	97	96	127	97.1
Urea (mmol/L)	3.51	31.7	22.6	24.5	12.9	5.38
Creatinine (μmol/L)	150.9	492	318	344	168.4	93.1

^a^ Date of the first hemodialysis.

The patient had 1 more session of plasmapheresis after remission due to financial constraints as against three proposed sessions and the discharge plan was to see the hematologist, neurologist, and physiotherapist on an outpatient basis. The plan also included a laboratory request to do ADAMTS‐13 assay. She was also counseled on the possibility of a relapse and other treatment methods such as steroids and rituximab for the relapse.

## 3 DISCUSSION

TTP is a hematological emergency that requires prompt therapy to reduce morbidity and mortality. TTP is characterized by the deposition of intravascular platelet microthrombi induced by autoantibody‐mediated deficiency of ADAMTS13, a von Willebrand factor (VWF)‐cleaving protease,^8^ resulting in thrombocytopenia, microangiopathic hemolytic anemia (MAHA), renal abnormalities, neurologic disturbances, and fever. The pentad of clinical syndromes which was proposed to constitute the diagnosis of TTP only occurs in 5% of cases. Currently, clinical diagnosis is made when there is the presence of microangiopathic hemolytic anemia, and thrombocytopenia, with or without neurological and renal involvement and another identifiable cause.^9^ Our patient presented with stroke, seizures, intracerebral hemorrhage, MAHA, thrombocytopenia, and acute kidney injury. There was no identifiable cause in our patient. TTP may also present with a transient ischemic attack (TIA) or stroke with or without hematological changes.^10^, ^11^ In a series of 47 patients with acute TTP, the most common neuroradiologic finding was posterior reversible encephalopathy syndrome (PRES), while large ischemic infarctions and hemorrhage were uncommon.^12^ Our patient presented with an ischemic stroke and later intracerebral hemorrhage occurred. Many patients with TTP present with the triad of thrombocytopenia, microangiopathic hemolysis, and neurological abnormalities. Some of them may also have a fever and renal abnormalities. However, it is important to note that neither the triad nor the pentad of the presentation can be relied upon for the diagnosis of TTP. In practice, a constellation of thrombocytopenia and microangiopathic hemolysis should always raise suspicion of TTP. Acute kidney injuries requiring dialysis are uncommon^13^ but our patient had an acute kidney injury and was hemodialysed while undergoing a plasma exchange transfusion.

TTP results from either a congenital or acquired decrease/absence of the von Willebrand factor protease ADAMTS13. Low levels of ADAMTS13 result in microthrombi formation which leads to end‐organ ischemia or damage.^14^, ^15^, ^16^ This is due to the inability of the ADAMTS13 to cause inactivation of the large multimer von Willebrand factor (vWF) that is necessary to prevent spontaneous coagulation. These larger multimers have a high avidity to bind platelets and initiate thrombi formation. However, the availability of ADAMTS13 activity assays is not available in many developing countries,^17^ making the confirmation of the diagnosis difficult. In attempting to solve this problem, prognostic scores are now being used to reduce the chance of mistakes and increase clinical diagnosis accuracy. Among these developed scores is the PLASMIC score which is practical and effective, according to some validated studies.^17^, ^18^ Since our patient could not afford the ADAMTS13 assay, and this assay is not readily available in the country, the PLASMIC score was used as an alternative to confirming our diagnosis of TTP. She had a risk score of 7 which is categorized as a high‐risk for TTP and a 96.2% risk of severe ADAMTS13 deficiency (≤10%) (Tables 3 and 4).

**TABLE 3:** PLASMIC score

Parameters	Plasmic score	
	Results	Score
Platelet count	18	1
Creatinine	150.9	1
INR	1.16	1
MCV	76.4	1
Presence of hemolysis variable	Raised LDH (2.6111 μ/L)	1
	Raised indirect bilirubin (34.7 μmol/L)	
History of stem cell or solid organ transplant	Nil	1
Active cancer	Nil	1

**TABLE 4:** Risk of severe ADAMTS‐13 deficiency score

Score	Risk category	Risk of severe ADAMTS‐13 deficiency (≤10%)
0–4	Low	4.3%
5–6	Intermediate	56.8%
7	High	96.2%

Measuring ADAMTS‐13 activity levels may not guarantee initial diagnosis and therapeutic decisions, but it is important for the prognosis. An ADAMTS‐13 activity level of <5%–10% seems to have increased specificity for TTP, but it does not identify all patients at risk for relapsing. ADAMTS‐13 activity levels of >10% make the diagnosis of TTP unlikely in patients presenting with acute thrombocytopenia.^10^, ^19^, ^20^ Sometimes transfusion of fresh frozen plasma may increase ADAMTS‐13 activity levels which may alter the diagnosis.

Therapeutic plasma exchange is the main therapy for patients with TTP.^7^ Plasma exchange delivers elevated ADAMTS‐13 dose without circulatory overload and removes antibodies to ADAMTS‐13, recovering ADAMTS‐13 activity. The treatment approach consists of a 1–1.5 plasma volume exchange with plasma daily until clinical symptoms have resolved and the platelet count has reached a normal level.^20^ Our patient's condition resolved after undergoing 7 sessions of plasmapheresis. LDH levels should also be monitored because it reflects ongoing tissue ischemia as well as hemolysis. Treatment with immunosuppressive agents is reserved for patients suspected of having ADAMTS‐13 autoimmune deficiency. Glucocorticoids are the immunosuppressive agents initially administered. Other agents such as rituximab and cyclosporine are used for more critically ill patients and patients with recurrent disease.^21^, ^22^

## 4 CONCLUSION

This case report shows how TTP can present as an ischaemic stroke which is an atypical presentation. It is interesting to note that our patient is the first Ghanaian to undergo plasmapheresis and the only case described in the literature and this makes our reported case a unique one. This may be due to late or missed diagnosis, the unavailability of screening tools, or inadequate funds to patronize plasma exchange. Rapid diagnosis and treatment are necessary for decreasing the risk of fatal outcomes in patients with TTP. This case illustrates the potential of TTP masquerading as ischemic stroke and it is necessary that clinicians are familiar with the clinical presentation and laboratory abnormalities of TTP, to make early diagnosis and initiate appropriate therapy. The diagnosis of stroke with TTP is atypical but a physician should suspect TTP in the setting of a stroke when the patient is a young female (two‐thirds of individuals with TTP are women), those who come with abnormal uterine bleeding without any identifiable cause, intracranial bleeds, petechiae and purpuric patches on the skin, and two prior episodes of stroke events.

## AUTHOR CONTRIBUTIONS

**Kwabena Oteng Agyapong:** Conceptualization; formal analysis; investigation; methodology; project administration; supervision; validation; visualization; writing – original draft; writing – review and editing. **Aba Folson:** Conceptualization; investigation; methodology; resources; supervision; writing – original draft; writing – review and editing. **Roland Wonkyi:** Conceptualization; investigation; methodology; supervision; visualization; writing – review and editing. **Kelvin Amenyedor:** Conceptualization; investigation; methodology; validation; writing – original draft; writing – review, and editing. **Jeffrey J. Boateng:** Investigation; methodology; writing – review, and editing. **Kate Fiador:** Investigation; supervision; writing – review, and editing.

## FUNDING INFORMATION

There has been no significant financial support for this work.

## CONFLICT OF INTEREST

The authors declare that they have no conflict of interest.

## CONSENT

Written informed consent was obtained from the patient to publish this report in accordance with the journal's patient consent policy. A copy of the written consent is available for review by the Editor‐in‐Chief of this journal.

## Data Availability

All data generated or analyzed during this study are included in this published article.
